# Supplementary material for: Spatiotemporal dynamics of habitat suitability for the Ethiopian staple crop, Eragrostis tef (teff), under changing climate
Source: PeerJ. 2021 Mar 22;9:e10965. doi: 10.7717/peerj.10965 (PMC7996070; doi:10.7717/peerj.10965)
Supplement: Supplemental Information 1 [file peerj-09-10965-s001.doc]

| **Bio1** | **1.00** |  |  |  |  |  |  |  |  |  |  |  |  |  |  |  |  |  |  |
| --- | --- | --- | --- | --- | --- | --- | --- | --- | --- | --- | --- | --- | --- | --- | --- | --- | --- | --- | --- |
| **Bio2** | -0.08 | 1.00 |  |  |  |  |  |  |  |  |  |  |  |  |  |  |  |  |  |
| **Bio3** | -0.42 | 0.23 | 1.00 |  |  |  |  |  |  |  |  |  |  |  |  |  |  |  |  |
| **Bio4** | 0.32 | -0.13 | -0.88 | 1.00 |  |  |  |  |  |  |  |  |  |  |  |  |  |  |  |
| **Bio5** | 0.97 | 0.07 | -0.49 | 0.42 | 1.00 |  |  |  |  |  |  |  |  |  |  |  |  |  |  |
| **Bio6** | 0.96 | -0.26 | -0.29 | 0.21 | 0.90 | 1.00 |  |  |  |  |  |  |  |  |  |  |  |  |  |
| **Bio7** | 0.23 | 0.71 | -0.52 | 0.54 | 0.42 | -0.02 | 1.00 |  |  |  |  |  |  |  |  |  |  |  |  |
| **Bio8** | 0.97 | -0.15 | -0.45 | 0.32 | 0.92 | 0.92 | 0.19 | 1.00 |  |  |  |  |  |  |  |  |  |  |  |
| **Bio9** | 0.97 | -0.13 | -0.37 | 0.27 | 0.94 | 0.96 | 0.15 | 0.92 | 1.00 |  |  |  |  |  |  |  |  |  |  |
| **Bio10** | 0.99 | -0.09 | -0.52 | 0.45 | 0.98 | 0.93 | 0.30 | 0.96 | 0.96 | 1.00 |  |  |  |  |  |  |  |  |  |
| **Bio11** | 0.99 | -0.05 | -0.29 | 0.16 | 0.94 | 0.96 | 0.16 | 0.95 | 0.97 | 0.96 | 1.00 |  |  |  |  |  |  |  |  |
| **Bo12** | -0.63 | 0.09 | 0.33 | -0.22 | -0.56 | -0.55 | -0.14 | -0.74 | -0.53 | -0.61 | -0.61 | 1.00 |  |  |  |  |  |  |  |
| **Bio13** | -0.57 | 0.34 | 0.17 | -0.06 | -0.43 | -0.56 | 0.18 | -0.69 | -0.50 | -0.53 | -0.56 | 0.85 | 1.00 |  |  |  |  |  |  |
| **Bio14** | -0.47 | -0.19 | 0.40 | -0.27 | -0.51 | -0.35 | -0.43 | -0.47 | -0.39 | -0.49 | -0.45 | 0.60 | 0.22 | 1.00 |  |  |  |  |  |
| **Bio15** | 0.25 | 0.46 | -0.12 | 0.05 | 0.35 | 0.15 | 0.47 | 0.21 | 0.19 | 0.26 | 0.27 | -0.30 | 0.16 | -0.66 | 1.00 |  |  |  |  |
| **Bio16** | -0.56 | 0.28 | 0.19 | -0.08 | -0.44 | -0.54 | 0.12 | -0.69 | -0.48 | -0.52 | -0.55 | 0.91 | 0.98 | 0.29 | 0.05 | 1.00 |  |  |  |
| **Bio17** | -0.51 | -0.21 | 0.43 | -0.31 | -0.56 | -0.39 | -0.47 | -0.49 | -0.43 | -0.53 | -0.49 | 0.60 | 0.19 | 0.96 | -0.75 | 0.26 | 1.00 |  |  |
| **Bio18** | -0.59 | 0.03 | 0.45 | -0.46 | -0.64 | -0.57 | -0.29 | -0.53 | -0.58 | -0.64 | -0.55 | 0.47 | 0.18 | 0.60 | -0.49 | 0.22 | 0.68 | 1.00 |  |
| **Bio19** | -0.26 | 0.14 | 0.11 | 0.00 | -0.16 | -0.20 | 0.06 | -0.42 | -0.14 | -0.22 | -0.24 | 0.78 | 0.77 | 0.24 | 0.02 | 0.83 | 0.19 | 0.03 | 1.00 |
|  | **Bio1** | **Bio2** | **Bio3** | **Bio4** | **Bio5** | **Bio6** | **Bio7** | **Bio8** | **Bio9** | **Bio10** | **Bio11** | **Bio12** | **Bio13** | **Bio14** | **Bio15** | **Bio16** | **Bio17** | **Bio18** | **Bio19** |

Table S1. Correlation of environmental variables. Variables Bio2, Bio3, Bio4, Bio7, Bio11, Bio14, Bio15, Bio18, and Bio19 were deemed independent and used in subsequent analyses.
